# Transcriptome and metabolome analyses reveal the regulatory role of *MdPYL9* in drought resistance in apple

**DOI:** 10.1186/s12870-024-05146-w

**Published:** 2024-05-24

**Authors:** Mingxiao Liu, Yitong Liu, Wei Hu, Baoying Yin, Bowen Liang, Zhongyong Li, Xueying Zhang, Jizhong Xu, Shasha Zhou

**Affiliations:** College of Horticulture, Hebei Agricultural University, Baoding, Hebei 071000 China

**Keywords:** Apple, Drought stress, Transcriptome, Metabolome, *MdPYL9*

## Abstract

**Background:**

The mechanisms by which the apple *MdPYL9* gene mediates the response to drought stress remain unclear. Here, transcriptome and metabolome analyses of apple plants under drought were used to investigate the mechanisms by which *MdPYL9* regulates the response to drought stress in apple. *MdPYL9-*overexpressed transgenic and non-transgenic apple histoculture seedlings were rooted, transplanted, and subjected to drought treatments to clarify the mechanisms underlying the responses of apples to drought stress through phenotypic observations, physiological and biochemical index measurements, and transcriptomic and metabolomic analyses.

**Results:**

Under drought stress treatment, transgenic plants were less affected by drought stress than non-transgenic plants. Decreases in the net photosynthetic rate, stomatal conductance, and transpiration rate of transgenic apple plants were less pronounced in transgenic plants than in non-transgenic plants, and increases in the intercellular CO_2_ concentration were less pronounced in transgenic plants than in non-transgenic plants. The relative electrical conductivity and content of malondialdehyde, superoxide anion, and hydrogen peroxide were significantly lower in transgenic plants than in non-transgenic plants, and the chlorophyll content and activities of antioxidant enzymes (superoxide dismutase, peroxidase, and catalase) were significantly higher in transgenic plants than in non-transgenic plants. The number of differentially expressed genes (DEGs) involved in the response to drought stress was lower in transgenic plants than in non-transgenic plants, and the most significant and highly annotated DEGs in the transgenic plants were involved in the flavonoid biosynthesis pathway, and the most significant and highly annotated DEGs in control plants were involved in the phytohormone signal transduction pathway. The number of differentially accumulated metabolites involved in the response to drought stress was lower in transgenic plants than in non-transgenic plants, and up-regulated metabolites were significantly enriched in apigenin-7-O-glucoside in transgenic plants and in abscisic acid in non-transgenic plants. In the flavonoid biosynthetic pathway, the expression of genes encoding chalcone synthase (CHS) and chalcone isomerase (CHI) was more significantly down-regulated in non-transgenic plants than in transgenic plants, and the expression of the gene encoding 4-coumarate-CoA ligase (4CL) was more significantly up-regulated in transgenic plants than in non-transgenic plants, which resulted in the significant up-regulation of apigenin-7-O-glucoside in transgenic plants.

**Conclusions:**

The above results indicated that the over-expression of *MdPYL9* increased the drought resistance of plants under drought stress by attenuating the down-regulation of the expression of genes encoding CHS and CHI and enhancing the up-regulated expression of the gene encoding 4CL, which enhanced the content of apigenin-7-O-glucoside.

**Supplementary Information:**

The online version contains supplementary material available at 10.1186/s12870-024-05146-w.

## Introduction

Apple is one of the main cultivated fruit tree species worldwide. Apples from China account for more than half of the world’s apple-planting area and output; China is thus the world’s largest apple producer [1]. Abiotic stresses such as drought and salt stress have become the main factors limiting the growth and distribution of apple plants [2]. Most of the high-quality apple-producing areas in China are located in shallow hilly areas with low rainfall and limited water resources, which, coupled with the highly uneven distribution of water resources, results in frequent droughts. The area of arid and semi-arid arable land in China accounts for more than half of the total available crop area, and there is a temporal and spatial mismatch between natural precipitation and critical water demand periods in apple production areas in China, and this is responsible for serious losses to apple production [3]. Therefore, the use of molecular methods to improve the drought resistance of apples is critically important for increasing apple yield and promoting the development of the apple industry.

Abscisic acid (ABA) is an important plant hormone that plays a key role in the response to abiotic stress. Abiotic stress stimulates the synthesis and accumulation of endogenous ABA, and the ABA signal transduction mechanism converts ABA stimulation into physiological responses to promote adaptation to adverse environmental conditions [4]. Many regulatory factors have been identified in the ABA signaling pathway, including protein kinases, phosphatases, and transcription factors. After the discovery of the ABA receptors PYR/PYL/RCAR (PYLs) in Arabidopsis cells in 2009, several studies have examined ABA perception and signal transduction [5, 6]. Similar genes have been reported in tomato [7], rice [8], grape [9], soybean [10], and rapeseed [11]. The Arabidopsis PYL family consists of 14 members, AtPYR1 and AtPYL1–13. In protoplast transfection experiments, all members (except PYL13) can activate ABA response genes [12]. Arabidopsis PYL family members can be divided into two groups according to their oligomeric state. Some are dimers (PYR1, PYL1, PYL2, and PYL3), and some are monomers (PYL4, PYL5, PYL6, and PYL8) [13]. In the absence of ABA, monomeric receptors can interact with PP2C to mediate ABA-independent abiotic stress response pathways [14]. ABA is necessary for the two groups of receptors to inhibit PP2C activity [15].

The ABA core signaling pathway consists of three main components: ABA receptor PYL protein, negative regulator class A PP2Cs (2 C type protein phosphatase), and positive regulator *SnRK2s* [16]. Drought stimulates ABA synthesis; the ABA signal is perceived by the PYL protein, and PYL bound to ABA binds to PP2C to form the ABA-PYL-PP2C ternary complex, thereby inactivating PP2C and releasing SnRK2 from PP2C inhibition [17, 18]. SnRK2 can then be autophosphorylated and activated; active SnRK2 kinase phosphorylates downstream effectors, which induces stomatal closure and the expression of abiotic stress response genes [12].

ABA mediates the response of plants to drought by binding to PYL receptor proteins, which induces a series of countermeasures that enhance resistance [19]. PYL receptor proteins regulate stress-related biological responses in the ABA signaling pathway and play a key role in enhancing the drought resistance of plants [19]. Overexpression of the rice ABA receptor gene *PYL10* increases ABA levels in plants due to the up-regulated expression of ABA biosynthesis genes (including *ZEP1, NCED1, NCED2, NCED3*, and *NCED4*). The expression of *PYL10* has been shown to increase yield under drought stress by maintaining a higher RWC, membrane stability index, and chlorophyll content and a low accumulation of malondialdehyde (MDA) and hydrogen peroxide (H_2_O_2_) content [20]. Overexpression of *OsPYL6* increased the total root length of seedlings, enhanced the accumulation of ABA in plants, significantly reduced the transpiration of plants, and actively regulated the expression of stress response genes and the dehydration resistance of plants [21]. However, the overexpression of *PYL* genes can induce dysplasia. For example, the overexpression of *OsPYL5* leads to reduced rice yield, and the overexpression of *AtPYL4* and its homologous genes in tomato leads to smaller rosette leaves in *Arabidopsis thaliana* [22]. The overexpression of *PYL9* increased the drought resistance of Arabidopsis and rice but promoted leaf senescence [23].

Previous studies have shown that *MdPYL9*, a member of the *PYL* gene family in apple, is overexpressed in apple and the WUE and drought resistance of *MdPYL9*-overexpressing apple lines is higher than that of non-transgenic lines under drought stress [24]. Here, we conducted transcriptomic and metabolomic analyses of the leaves of *MdPYL9*-overexpressing apple lines and non-transgenic plants to investigate the regulatory mechanisms of *MdPYL9* for drought resistance. The results of this study provide insights into the pathways by which *MdPYL9* regulates drought resistance in apples and will aid the production of new apple germplasm with high drought resistance for cultivation in arid regions via genetic engineering.

## Materials and methods

### Plant materials

*MdPYL9*-overexpressing transgenic and non-transgenic GL3 apple histoculture plants.

### Rooting and transplanting of histoculture seedlings

The stem tips of histoculture apple seedlings, which had been grown for approximately 30 d, were cut off and inserted into the rooting medium. After 30 days of rooting, the roots of the seedlings were washed with distilled water and transferred to plastic pots (1.8 × 4.8 × 9.3 cm) containing a 3:1:1 ratio of soil, vermiculite, and perlite. After 30 days of incubation, seedlings were transplanted into plastic pots containing forest soil, sand, and organic substrate in a 5:1:1 ratio and grown in a greenhouse in 9.2 × 14 × 12 cm plastic pots. Plants were watered every 3 days and every 7 days with 1/2 concentration of Hoagland’s nutrient solution (pH 6.0). Approximately 200 rooted plants each of transgenic and non-transgenic apple plants were grown.

The specific components of the media were as follows: MS medium (4.43 g/L MS, 7.8 g/L agar, and 30 g/L sucrose); subculture medium (MS medium, 0.3 mg/L 6-BA, 0.2 mg/L IAA, and 0.1 mg/L GA3); rooting medium (2.22 g/L MS, 6 g/L agar, 20 g/L sucrose, and 0.4 mg/L IBA).

### Short-term drought treatment of apple plants

The short-term drought treatment for the experiment was conducted at the Innovation Experiment Park of Hebei Agricultural University. Normal-watered and short-term natural drought treatment: 60 non-transgenic apple plants and 60 transgenic apple plants of uniform growth were selected and randomly divided into two groups respecyively (normal-watered group and short-term natural drought treatment group). All plants was fully irrigated before starting the treatments. Normal-watered group: the treatment was done by supplying normal water during the period (maintain soil relative water content 75-85%); short-term natural drought group: stopping water supply after fully irrigation. It’s 0th day when the soil relative water content reached 75-85% after fully irrigated.

In general, this study includes the following four treatments:

1. WT-CK: Normal-watered non-transgenic GL3 apple plants (the relative water content of the soil was maintained at 75-85%);
2. WT-D: The short-term natural drought treatment non-transgenic apple plants;
3. OE-CK: Normal-watered *MdPYL9*-overexpressing transgenic apple plants(the relative water content of the soil was maintained at 75-85%).
4. OE-D: The short-term natural drought treatment *MdPYL9*-overexpressing transgenic apple plants.

### Sample and restoration of water supply (rehydration)

Samples were taken on the 6th day of the short-term drought treatment (significant differences were observed in the phenotypes of different treatment groups at this time), There were three replicates for each treatment, and leaves of 10 seedlings were collected from the same position per replicate (2–3 uniform, healthy, mature leaves from each plant, 9-11th leaves from bottom to top). The samples were immediately frozen in liquid nitrogen and stored at -80 °C in an ultra-low temperature refrigerator. After sample, all plants including the short-term natural drought treatment ones were then watered normally (maintain soil relative water content 75-85%).

### Determination of physiological and biochemical indicators

Measurement of photosynthetic parameters: The instrument used was LI-COR 6800 portable photosynthesis meter (Beijing Ligao Tai Science and Technology Co., Ltd.), and the photosynthetic parameters were measured at 9:00–11:00 am on a sunny day, and the net photosynthetic rate (Pn), stomatal conductance (Gs), intercellular CO_2_ concentration (Ci), and transpiration rate (Tr) of the mature leaves (9-11th leaves from bottom to top) were measured in five plants of the same length randomly selected for each treatment. REL was measured with a thundermagnetic DDS-307 conductivity meter [25]. Superoxide dismutase (SOD) activity was determined using the nitrogen blue tetrazolium (NBT) method [26]. Catalase (CAT) activity was determined using the ultraviolet (UV) absorption method [27]. Peroxidase (POD) activity was determined using the guaiacol method [28].The MDA content was determined using the thiobarbituric acid method [27]. The specific steps refer to the corresponding literatures. The superoxide anion content was determined following the method of Zhang [29]. The H_2_O_2_ content was determined using a kit (Suzhou GRS Biotechnology Co., Ltd.). The chlorophyll content was determined following the method of Yang [30]. The relative electrical conductivity (REL) was determined following the method described by Yang [30].

### Transcriptome sequencing and validation of target gene expression

Transcriptome analyses were performed on four leaf samples groups, including WT-CK, WT-D, OE-CK and OE-D (explanation as above).The total RNA of the apple leaves was separated using TRIzol reagent (Invitrogen, Carlsbad, CA, USA) for transcriptome analysis. After extraction of total RNA, Illumina RNA-Seq was performed by Metware Biotechnology Co. Ltd. (Wuhan, China). Purified RNA (1 µg each sample) was reverse transcribed to first-strand cDNA using the cDNA Reverse Transcription Kit (PrimeScript™ RT Master Mix, Takara Bio, Ohtsu, Japan) according to the manufacturer’s instructions. The raw reads were transformed from the raw sequencing image data using CASAVA base recognition. The adapter sequences were cut, and low-quality reads with ≥ 5 uncertain bases or with more than 50% 4 Qphred ≤ 20 bases were removed using fastp to obtain the high-quality data. The GC content of the clean reads was calculated. The Q20 and Q30 values were also determined by FastQC to evaluate base quality. Fragments per kilobase of transcript per million fragments mapped was calculated as an indicator to measure the transcripts or DEGs (Differentially Expressed Genes). DESeq2 is a suitable method for differential expression analysis between sample groups with biological replicates to obtain DEG sets between two biological conditions [31]. The conditions for allogeneic screening were |log2 (fold change)| ≥ 1 and a false discovery rate < 0.05. The DEGs were analyzed using gene ontology (GO) and the Kyoto Encyclopedia of Genes and Genomes (KEGG) tools [32].

Total RNA was extracted from each treated leaf using the M5 Plant RNeasy Complex Mini Kit (Mei5 Biotechnology Co., Ltd., Beijing, China), as directed by the manufacturer. The concentration, purity, and integrity of total RNA were measured using a NanoDrop 2000 spectrophotometer (Thermo Scientific, Wilmington, DE, USA). The inverse transcription was carried out using the UEIrisIIRT-PCR System for First-Strand cDNA Synthesis system (Suzhou US Everbright, Inc., Suzhou, China). The qRT-PCR reaction was performed using the TransStart® Top Green qPCR Super Mix (TransGen Biotech Co., Ltd., Beijing, China) and LightCycler® 96 Real-Time PCR System (Qiangxin Biorepublic Co., Ltd, Beijing, China).The 4-coumarate-CoA ligase *(4CL*) (MD07G1309000-*4CL2*), chalcone synthase (*CHS*) (MD13G1285100-*CHS1*, MD04G1003000-*CHS2*, MD04G1003300-*CHS5*, MD04G1003400-*CHS5 like*) and chalcone isomerase (*CHI*) genes (MD01G1167300, MD01G1233400) and 10 randomly selected DEGs (MD12G1078200, MD16G1131400, MD01G1167300, MD09G1137200, MD01G1040200, MD05G1209700, MD01G1167300, MD04G1167000, MD01G1202100, MD01G1217800, MD13G1148300) were validated by qRT-RCR. The primers for all genes are shown in Supplementary Table S1. Three replicates were set for each treatment, and the 2^−∆∆Ct^ method was used to analyze the normalized expression of each sample.

### Widely targeted metabolomics

Transcriptome analyses were performed on four leaf samples, including OE-CK, OE-D, WT-CK and WT-D.Sample preparation and extraction, metabolome profiling, and data analysis were performed according to the standard procedures of Wuhan MetWare Biotechnology Co., Ltd. (Wuhan, China) (www.metware.cn, accessed on 6 December 2020). The sample extracts were analyzed using an ultrahigh-performance liquid chromatography-electrospray ionization tandem mass spectrometry system (HPLC, Shimadzu Nexera X2, Kyoto, Japan, www.shimadzu.com.cn/, accessed on 6 December 2020; MS, Applied Biosystems 4500 Q TRAP, Carlsbad, CA, USA, www.appliedbiosystems.com.cn/, accessed on 6 December 2020). The metabolite data were log2-transformed to improve normality for the statistical analysis and were normalized. Principal component analysis (PCA) was carried out to preliminarily understand the overall metabolic differences among the samples in each group and the degree of variation among the samples within the group. Metabolites with variable importance in projection (VIP) ≥ 1.0, |log_2_(fold change)| ≥ 1 were defined as significantly changed metabolites (SCMs).

### Statistical analysis

Microsoft Excel was used to process the data. IBM SPSS Statistics 23.0 software was used to conduct statistical analyses. Duncan’s multiple range test was performed following one-way ANOVA (*p* < 0.05).

## Results

### Phenotypic changes in *MdPYL9*-overexpressing apple plants under drought

**Fig. 1:**
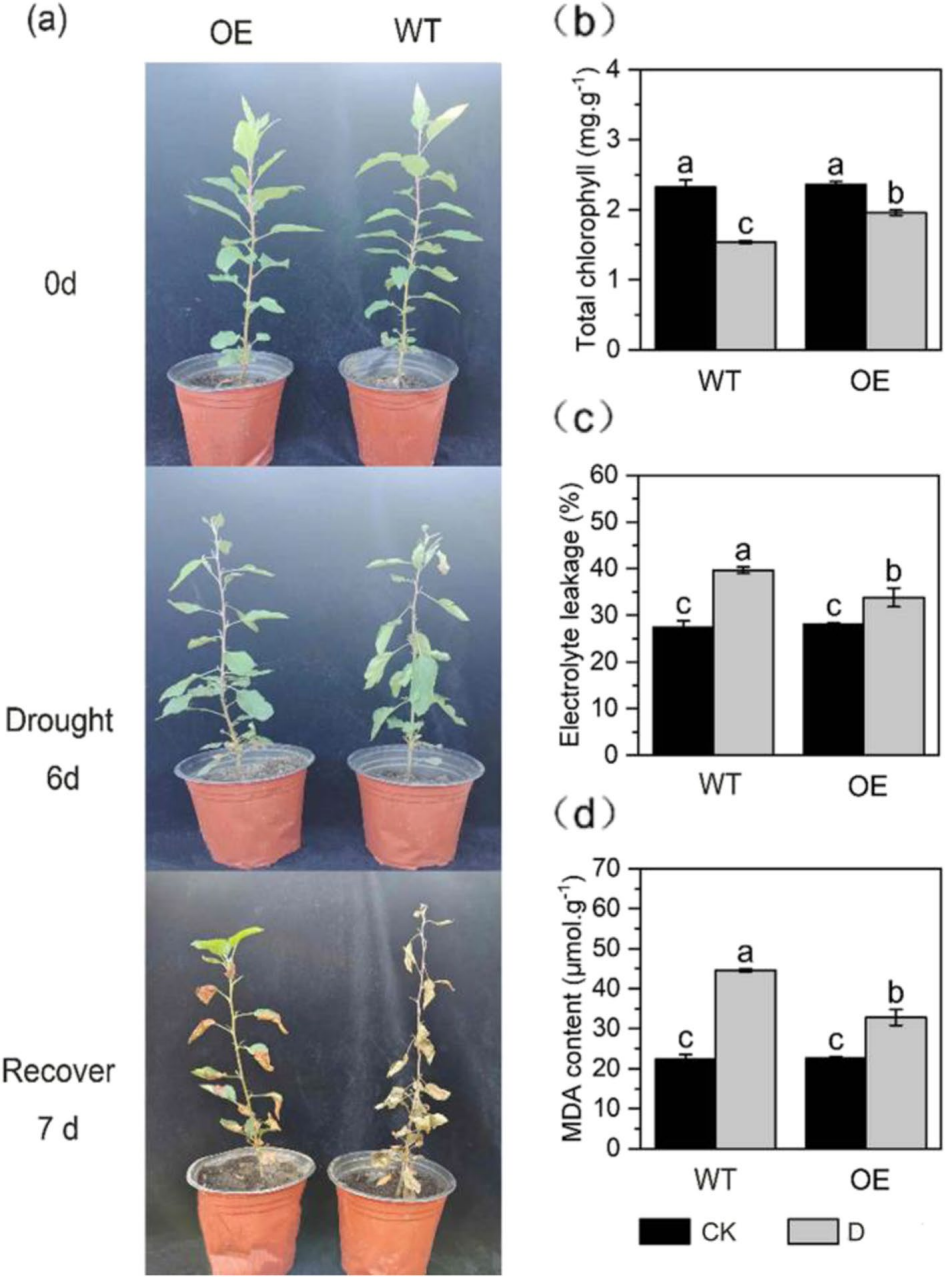
Phenotype, MDA content, chlorophyll content, and relative electrical conductivity of non-transgenic and transgenic plants under normal-watered and short-term natural drought stresses: **a** Phenotype of plants, **b** Chlorophyll content, **c** Relative electrical conductivity, **d** MDA content.Note: in the figures, ‘WT’ indicates non-transgenic apple plants, ‘OE’ indicates transgenic apple plants; ‘CK’ indicates normal-watered treatment, ‘D’ indicates short-term natural drought treatment. Data was the means of five replicates with SD. Bars labeled with different lowercase indicate significant differences at *P*< 0.05. The same belows

On the 6th d of the natural drought treatment, severe wilting was observed in non-transgenic plants, and wilting was less pronounced in transgenic plants. Non-transgenic apple plants dried and died after 7th day of rehydration, whereas the transgenic apple plants recovered to the normal growth state (Fig. 1a). Under normal-watered condition, the chlorophyll content, REL (relative electrical conductivity), and MDA content of all plants were roughly the same. After 6th days of drought treatment, the chlorophyll content of all plants decreased significantly. The decrease in the chlorophyll content of transgenic plants was significantly smaller than that of non-transgenic plants, and the chlorophyll content of transgenic plants was higher than that of non-transgenic plants (Fig. 1b). Under drought stress, the REL of all plants increased, and the increase in the REL was greater in non-transgenic plants than in transgenic plants (Fig. 1c). After drought stress, the MDA content of all plants increased significantly, but the MDA content of transgenic plants was significantly lower than that of non-transgenic plants (Fig. 1d).

### Analysis of the photosynthetic capacity

**Fig. 2:**
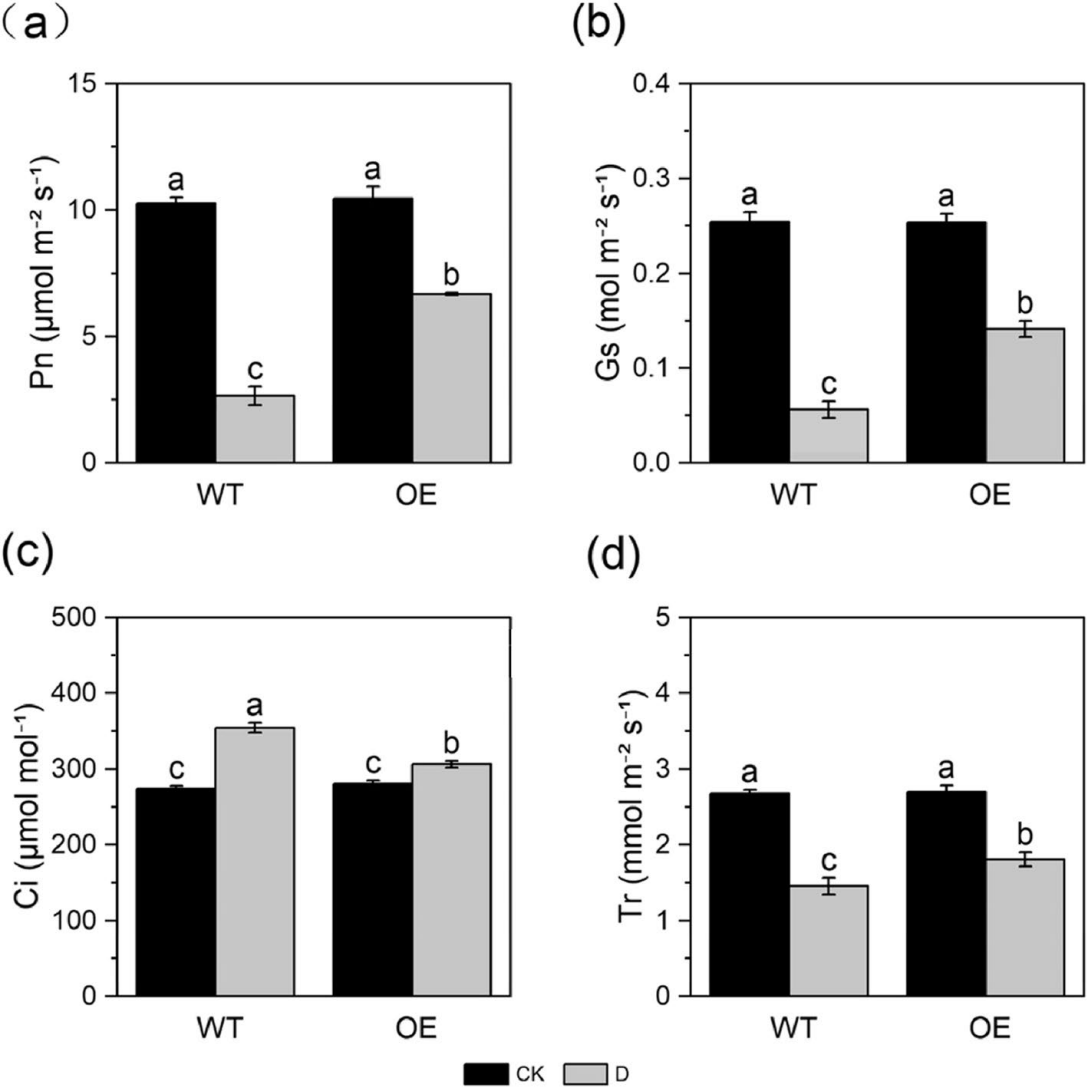
Photosynthetic parameters of non-transgenic and transgenic plants under normal-watered and short-term natural drought stresses: **a** net photosynthetic rate (Pn), **b** stomatal conductance (Gs), **c** intercellular CO_2_concentration (Ci), **d** transpiration rate (Tr)

Under normal-watered treatment, there was no significant difference in the net photosynthetic rate (Pn) between non-transgenic and transgenic apple plants. Under natural drought condition, the Pn of all plants decreased, but the Pn of transgenic apple plants was higher (Fig. 2a). Under normal-watered treatment, there was no difference in stomatal conductance (Gs) between non-transgenic and transgenic apple plants. Under natural drought conditions, the Gs of all plants decreased, but the Gs of transgenic apple plants was higher (Fig. 2b). This shows that stomatal opening was reduced in transgenic plants under natural drought conditions to cope with drought stress; this not only reduces water loss but also maintains high levels of photosynthesis, which mediates the responses of plants to stress. Under normal-watered treatment, there was no significant difference in the transpiration rate (Tr) between non-transgenic and transgenic apple plants. Under natural drought conditions, the Tr of all plants decreased, but the Tr of transgenic apple was higher (Fig. 2d). Under natural drought condition, the intercellular CO_2_ concentration (Ci) of all plants increased, but the Ci of transgenic apple plants increased less than that of non-transgenic plants (Fig. 2c). This is also consistent with the finding that the photosynthetic activity of transgenic apple plants was significantly greater than that of non-transgenic plants. Overall, the above photosynthetic data indicated that *MdPYL9*-overexpressing transgenic apple plants were able to maintain a higher photosynthetic capacity under natural drought treatment compared with non-transgenic plants.

### Analysis of antioxidant enzyme activity and the reactive oxygen species (ROS) content

**Fig. 3:**
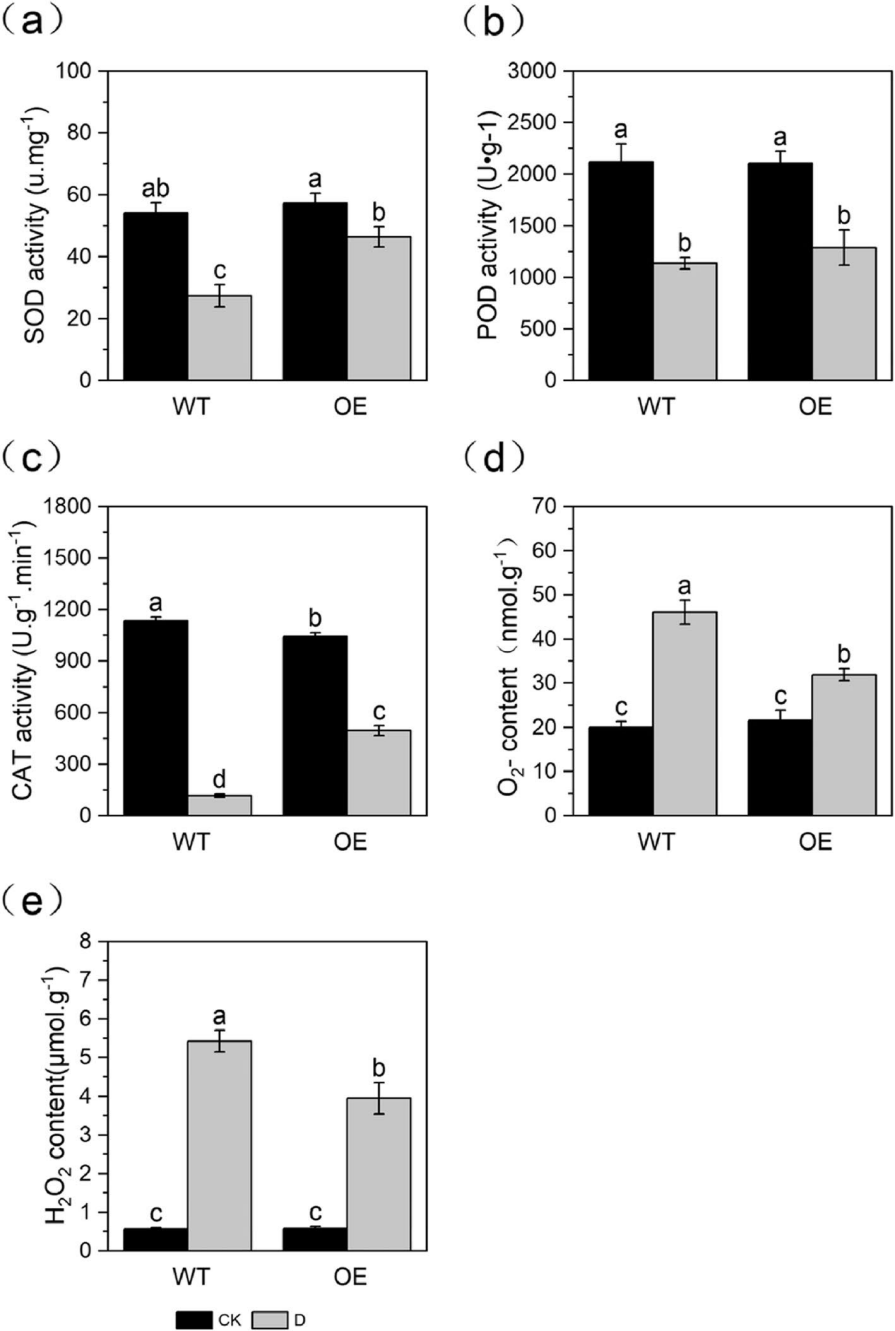
Analysis of antioxidant enzyme activity and the ROS content in leaves under normal-watered and short-term natural drought stresses: **a** SOD enzyme activity, **b** POD enzyme activity, **c** CAT enzyme activity, **d** O_2_^-^ content, **e** H_2_O_2_ content

After 6 d of drought stress, the SOD activity of both non-transgenic and transgenic plants significantly decreased, but the SOD activity of transgenic plants was significantly higher than that of non-transgenic plants (Fig. 3a). After 6 d of drought stress, the POD activity of both non-transgenic and transgenic plants was significantly reduced. But the POD activity of transgenic apple plants was not significantly different from that of non-transgenic plants both under normal-watered and drought condition (Fig. 3b). After 6 d of drought stress, the CAT activity of both non-transgenic and transgenic plants decreased significantly, but the CAT activity of non-transgenic plants decreased more than that of transgenic plants (Fig. 3c). Under normal-watered condition, the superoxide anion (O_2_^−^) and H_2_O_2_ content of transgenic and non-transgenic apple plants did not significantly differ. At 6 d of natural drought stress, the O_2_^−^ and H_2_O_2_ content of both transgenic and non-transgenic apple plants significantly increased, but the O_2_^−^ and H_2_O_2_ content was lower in transgenic plants than in non-transgenic plants (Fig. 3d-e).

### Transcriptome analysis and screening of DEGs (differentially expressed genes)

**Fig. 4:**
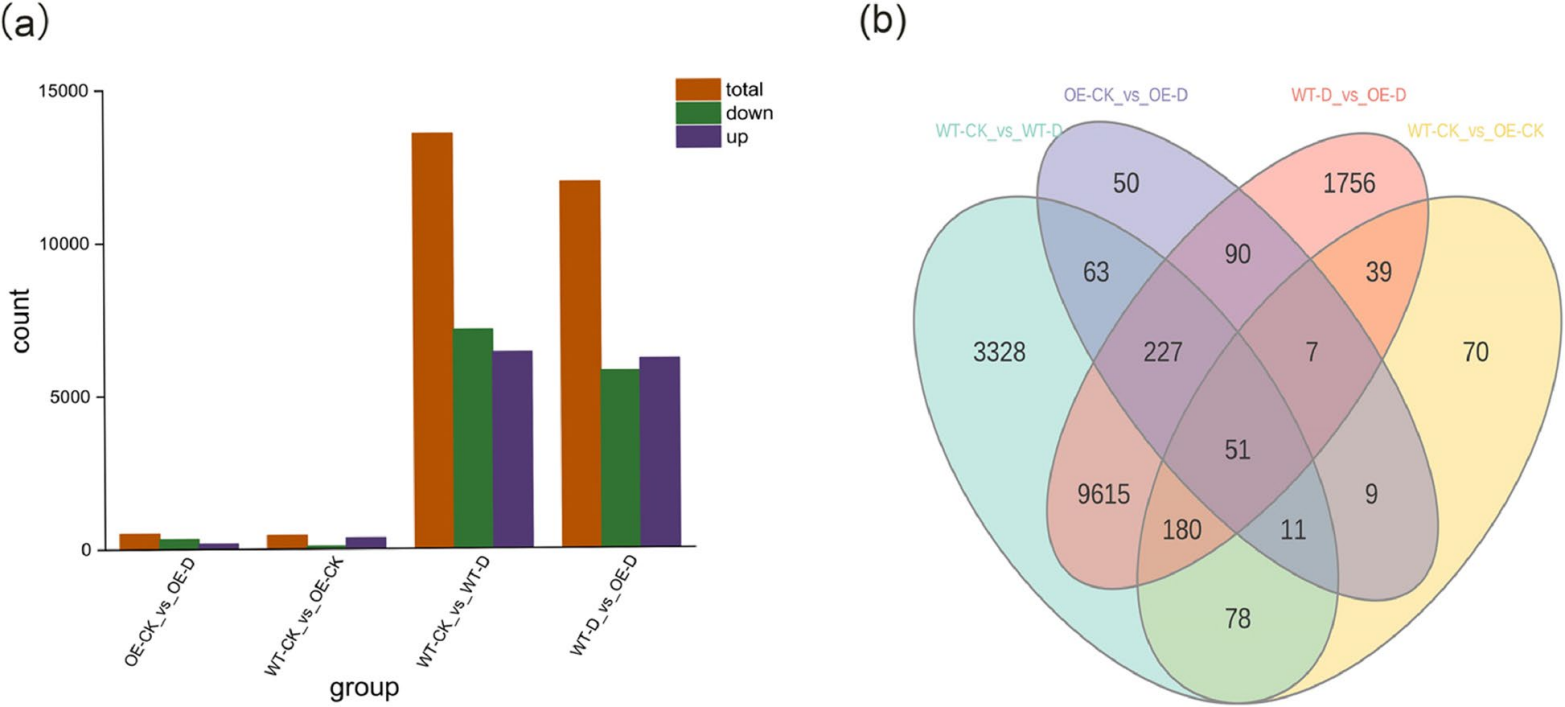
Analysis of DEGs (Differentially Expressed Genes) in leaves under normal-watered and short-term natural drought stresses: **a** Number of differential genes among different treatment groups, **b** Venn diagram for numbers of DEGs in the OE-CK_vs_OE-D、WT-CK_vs_OE-CK、WT-CK_vs_WT-D and WT-D_vs_OE-D comparisons

Transcriptome analysis was completed using RNA sequencing (RNA-Seq) technology, and a total of 82.85 Gb Clean Data were obtained, with each sample having at least 6 Gb Clean Data; the GC content ranged from 44.83 to 46.02, the Q20 base percentage was 97.67% and above, and the Q30 base percentage was 93.36% and above; these findings indicate that the quality of the sequencing data was high (Supplementary Table S2). DEGs were identified using the following criteria: |log2FoldChange| ≥1 and false discovery rate < 0.05. The total number of DEGs, the number of up-regulated genes, and the number of down-regulated genes in each group were determined after completing the analysis of DEGs using DESeq2/edgeR. As shown in Fig. 4a, a total of 508 DEGs (334 up-regulated and 174 down-regulated) were identified in transgenic plants in the normal-watered and short-term drought treatment groups (OE-CK_vs_OE-D) (Supplementary Table S3). A total of 13,553 DEGs (7,153 up-regulated and 6,400 down-regulated) were identified in non-transgenic plants under normal-watered and drought conditions (WT-CK_vs_WT-D) (Supplementary Table S4). A total of 445 DEGs (88 up-regulated and 357 down-regulated) were identified between transgenic and non-transgenic plants under normal-watered conditions (WT-CK_vs_OE-CK) (Supplementary Table S5). A total of 11,965 DEGs were identified in transgenic and non-transgenic plants under drought treatment (WT-D_vs_OE-D) (Supplementary Table S6), including 5,783 up-regulated genes and 6,182 down-regulated genes. There were 352 DEGs in transgenic plants in the normal-watered and drought treatment group vs. non-transgenic plants in the normal-watered and drought treatment groups. A total of 277 DEGs were identified in transgenic and non-transgenic plants in the normal-watered treatment group vs. transgenic and non-transgenic plants in the drought treatment group (Fig. 4b). All these results suggest that *MdPYL9* plays a regulatory role under drought stress.

### Functional analysis of DEGs

**Fig. 5:**
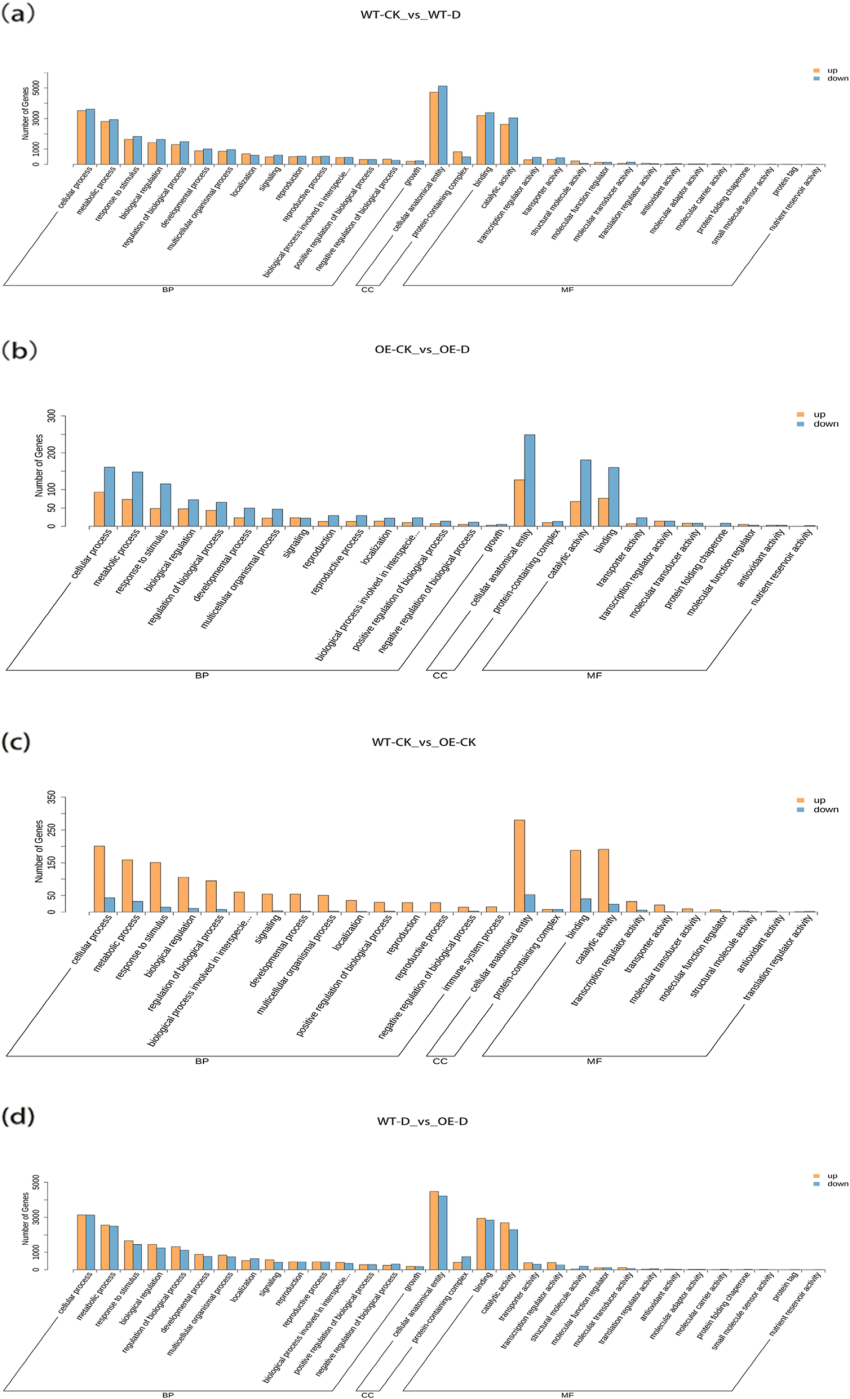
GO (Gene Ontology) classification of DEGs: **a** GO classification of DEGs in transgenic lines (WT-CK vs WT-D), **b** GO classification of DEGs in non-transgenic lines (OE-CK vs OE-D), **c** GO classification of DEGs under normal watered conditions (WT-CK vs OE-CK), **d** GO classification of DEGs under drought conditions (WT-D vs OE-D)

To verify the biological functions of the DEGs in transgenic and non-transgenic plants under drought stress treatment, Gene Ontology (GO) and Kyoto Encyclopedia of Genes and Genomes (KEGG) enrichment analyses were performed on the DEGs. The results of GO enrichment analysis (Fig. 5) showed that the main GO terms of the DEGs in the biological process category in transgenic plants were Cellular process (20.94%), Metabolic process (14.97%), Response to stimulus (10.41%), Biological regulation (9.07%), and Regulation of biological process (8.22%). The DEGs in the cell component category were mainly involved in Cellular anatomical entity (88.10%). The main enriched GO terms of DEGs in the molecular function category were Catalytic activity (38.04%) and Binding (44.19%). The results of the GO enrichment analysis of transgenic plants and non-transgenic plants were similar. The DEGs of non-transgenic plants were also mainly enriched in these GO terms, but the number of DEGs enriched in these GO terms was greater in non-transgenic plants than in transgenic plants. Non-transgenic plants might be more sensitive to drought and affected by drought stress.

The GO terms of differentially expressed genes in biological processes under normal treatment conditions were mainly enriched in cellular process (19.77%), metabolic process (5.48%), response to stimulus (13.45%), biological regulation (9.48%), regulation of biological process (8.35% ). The main enriched GO terms of differential genes in cell components were cellular anatomical entity (95.42%), and the main enriched GO terms of differential genes in molecular functions were binding (42.70%) and catalytic activity (40.26%). The enriched GO items under drought conditions were the same as those under normal treatment conditions, but these under drought conditions.

The results of KEGG enrichment analysis showed that there were (q value < 0.05) 38 pathways significantly enriched in non-transgenic plants in the normal-watered and drought treatment groups, including Carbon fixation in photosynthetic organisms, Photosynthesis-antenna proteins, Plant hormone signal transduction, and Photosynthesis. The greatest number of DEGs were involved in Metabolic pathways (1,848 genes), Biosynthesis of secondary metabolites (1,120 genes), and Plant hormone signal transduction pathway (520 genes); the Phytohormone signaling pathway was significantly enriched, and a high number of DEGs were annotated to this pathway (Supplementary Table S7). A total of 19 pathways were significantly enriched in transgenic plants in the normal-watered vs. drought treatment subgroups (q value < 0.05), including Flavonoid biosynthesis, Protein processing in endoplasmic reticulum, Biosynthesis of secondary metabolites, and Metabolic pathways. The greatest number of DEGs were involved in Metabolic pathways (121 genes), Biosynthesis pathways of secondary metabolites (90 genes), Protein processing in endoplasmic reticulum (34 genes), and Flavonoid biosynthesis pathways (25 genes). The Flavonoid biosynthesis pathway and Protein processing in endoplasmic reticulum were significantly enriched, and a high number of DEGs were annotated to these pathways (Supplementary Table S8).

### Validation of target genes by qRT-PCR

**Fig. 6:**
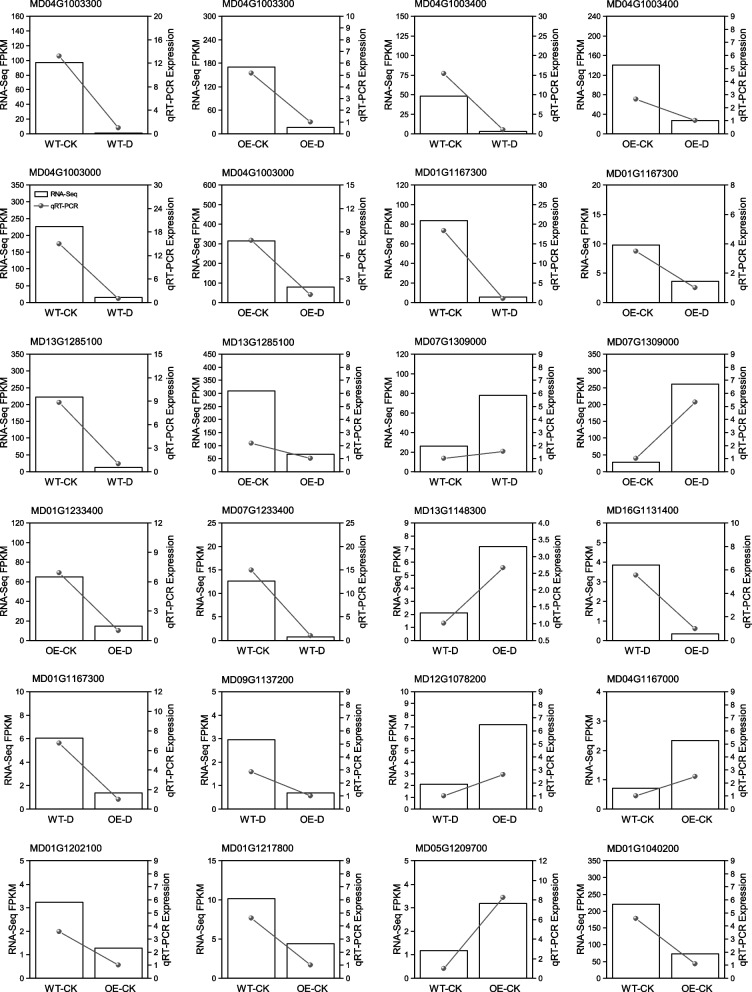
Transcriptome qRT-PCR validation, including one* 4CL* gene (MD07G1309000-*4CL2*), four *CHS* genes (MD13G1285100-*CHS1*, MD04G1003000-*CHS2*, MD04G1003300-*CHS5*, MD04G1003400-*CHS5 like*) and two *CHI* genes (MD01G1167300, MD01G1233400) and 10 randomly selected differentially expressed genes (MD12G1078200, MD16G1131400, MD01G1167300, MD09G1137200, MD01G1040200, MD05G1209700, MD01G1167300, MD04G1167000, MD01G1202100, MD01G1217800, MD13G1148300)

To verify the accuracy of the RNA-Seq data, 24 DEGs were randomly selected for qRT-RCR analysis. The expression patterns of these genes inferred by qRT-PCR were roughly similar to those inferred from the RNA-Seq data, indicating that the transcriptome data were accurate and reliable (Fig. 6).

#### Overview of the metabolites

**Fig. 7:**
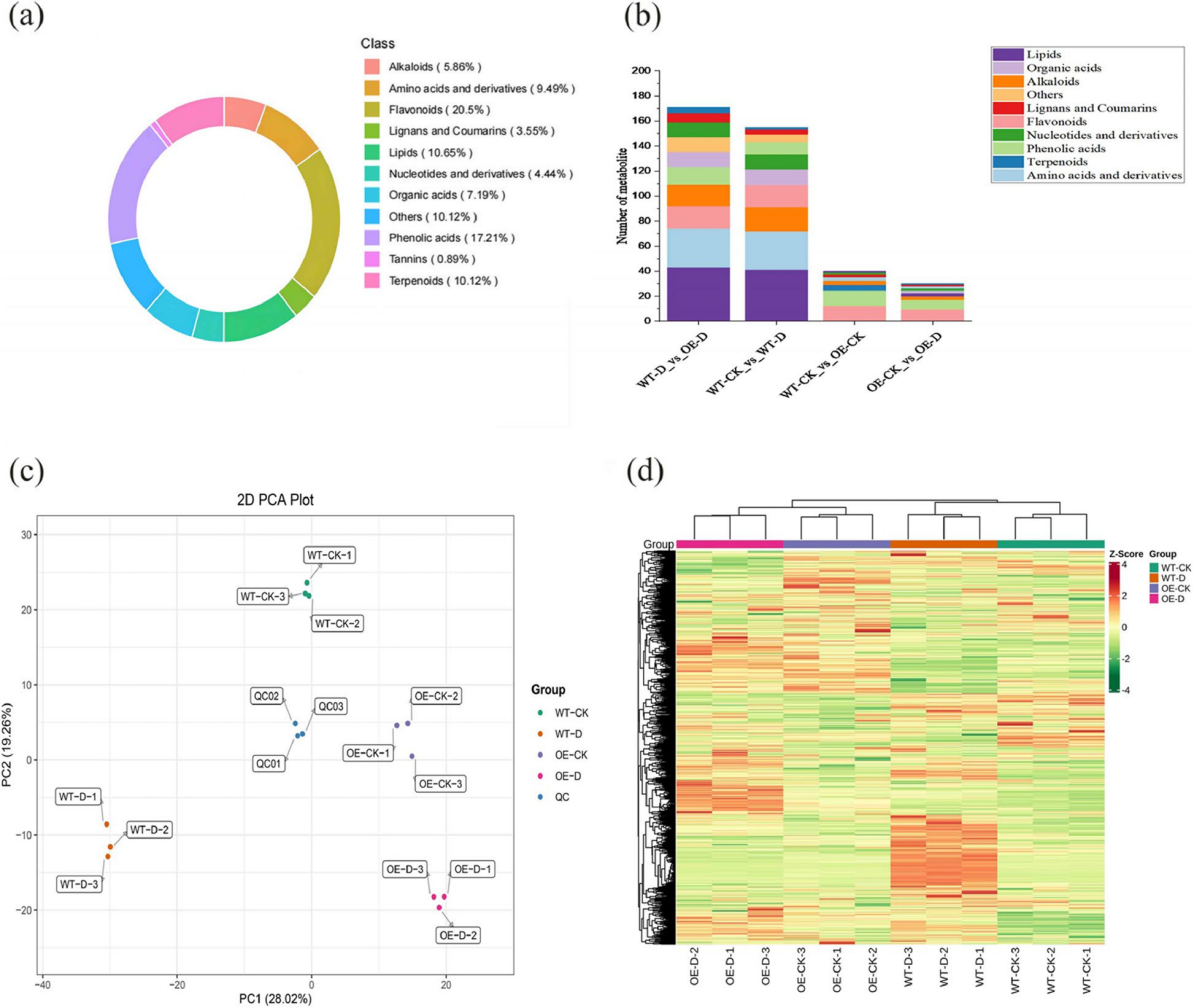
Qualitative and quantitative analysis of the metabolomics data: **a** Number of different types of metabolites, **b** Number of different types of metabolites in each treatment group, **c** Principal component analysis (PCA), **d** correlation heat map

To clarify changes in metabolites among treatments, primary metabolites and secondary metabolites in the samples were identified by a UPLC-MS/MS platform. A total of 1,127 metabolites were detected, including 107 Amino acids and derivatives, 114 Terpenoids, 194 Phenolic acids, 50 Nucleotides and derivatives, 23 Flavonoids, 40 Lignans and Coumarins, 114 Others, 10 Tannins, 66 Alkaloids, 81 Organic acids, and 120 Lipids. In transgenic and non-transgenic plants in the normal-watered treatment group, 40 metabolites were detected, including 3 Amino acids and derivatives, 5 Terpenoids, 12 Phenolic acids, 2 Nucleotides and derivatives, 12 Flavonoids, 2 Lignans and Coumarins, 3 Alkaloids, and 1 Lipid. In the normal-watered and drought treatment groups of transgenic lines, 30 metabolites were detected, including 2 Amino acids and derivatives, 1 Terpenoid, 8 Phenolic acids, 2 Nucleotides and derivatives, 9 Flavonoids, 1 Other, 3 Alkaloids, 2 Organic acids, and 2 Lipids. A total of 155 metabolites were detected in non-transgenic plants in the normal-watered and drought-treated groups, including 31 Amino acids and derivatives, 2 Terpenoids, 10 Phenolic acids, 12 Nucleotides and derivatives, 18 Flavonoids, 4 Lignans and Coumarins, 6 Others, 19 Alkaloids, 12 Organic acids, and 41 Lipids. A total of 171 metabolites were detected in transgenic and non-transgenic plants in the drought-treated group, including 31 Amino acids and derivatives, 5 Terpenoids, 14 Phenolic acids, 12 Nucleotides and derivatives, 18 Flavonoids, 7 Lignans and Coumarins, 12 Others, 17 Alkaloids, 12 Organic acids, and 43 Lipids (Fig. 7).

PCA can be used to capture variation in metabolomic characteristics via several principal components, and differences between groups can be observed in PCA plots. Treatments were clearly separated in the PCA plot, which indicates that the treatments induced significant changes in metabolites in the samples; this is consistent with the observed phenotypes (physiological indicators). The first principal component (PC1) explained 28.02% of the variation in the original dataset, and separation between transgenic and non-transgenic plants was observed; the drought treatment was correlated with the second principal component, which explained 19.26% of the variation in the original dataset (Fig. 7a). To further analyze the homogeneity among biological replicates within the samples, we normalized the content of all the measured metabolites, conducted a cluster analysis, and generated a heat map (Fig. 7b). The three biological replicates of the normal-watered and drought-treated non-transgenic and transgenic plants were clustered into one category each, indicating that the intra-group gap between these three biological replicates was small.

### Analysis of differentially accumulated metabolites (DAMs)

**Fig. 8:**
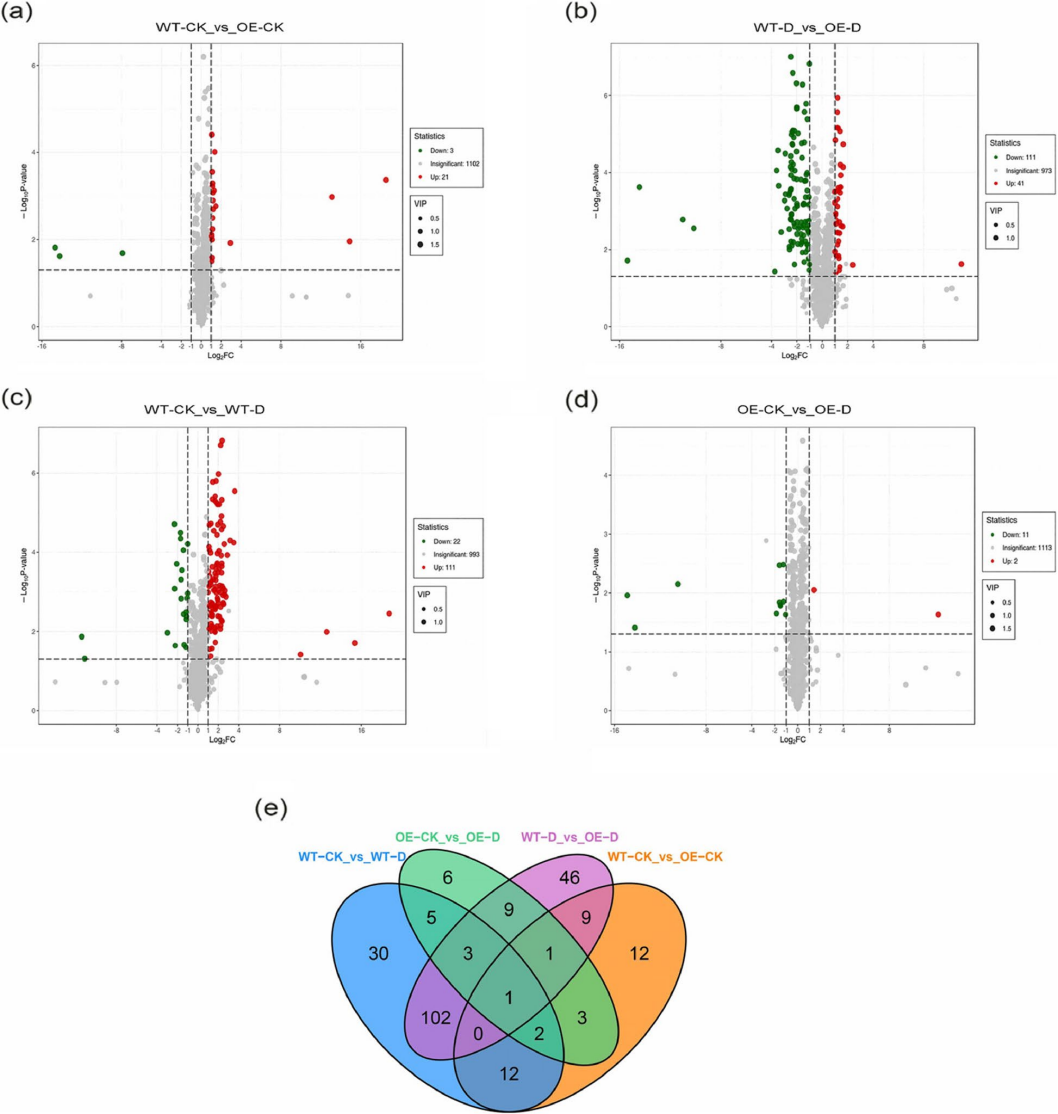
Volcano plot and Venn diagram of DAMs (differentially accumulated metabolites) among groups: **a**-**d** Volcano plots of DAMs among groups, **e** Venn diagrams of DAMs among groups

A total of 41 up-regulated and 111 down-regulated metabolites were identified in transgenic plants in the normal-watered and drought-treated groups; 21 up-regulated and 3 down-regulated metabolites were identified in non-transgenic plants in the normal-watered and drought-treated groups. In transgenic and non-transgenic plants in the normal-watered treatment group, 111 metabolites were up-regulated, and 22 were down-regulated. In transgenic and non-transgenic plants in the drought-treated group, 11 metabolites were up-regulated, and 2 were down-regulated (Fig. 8a-d). A Venn diagram of DAMs revealed that there were 11 common DAMs in transgenic and non-transgenic plants in the drought-treated group and transgenic and non-transgenic plants in the normal-treated group; there were 11 common DAMs in transgenic plants in the normal-watered and drought-treated groups and in non-transgenic plants in the normal-watered and drought-treated groups (Fig. 8e).

**Fig. 9:**
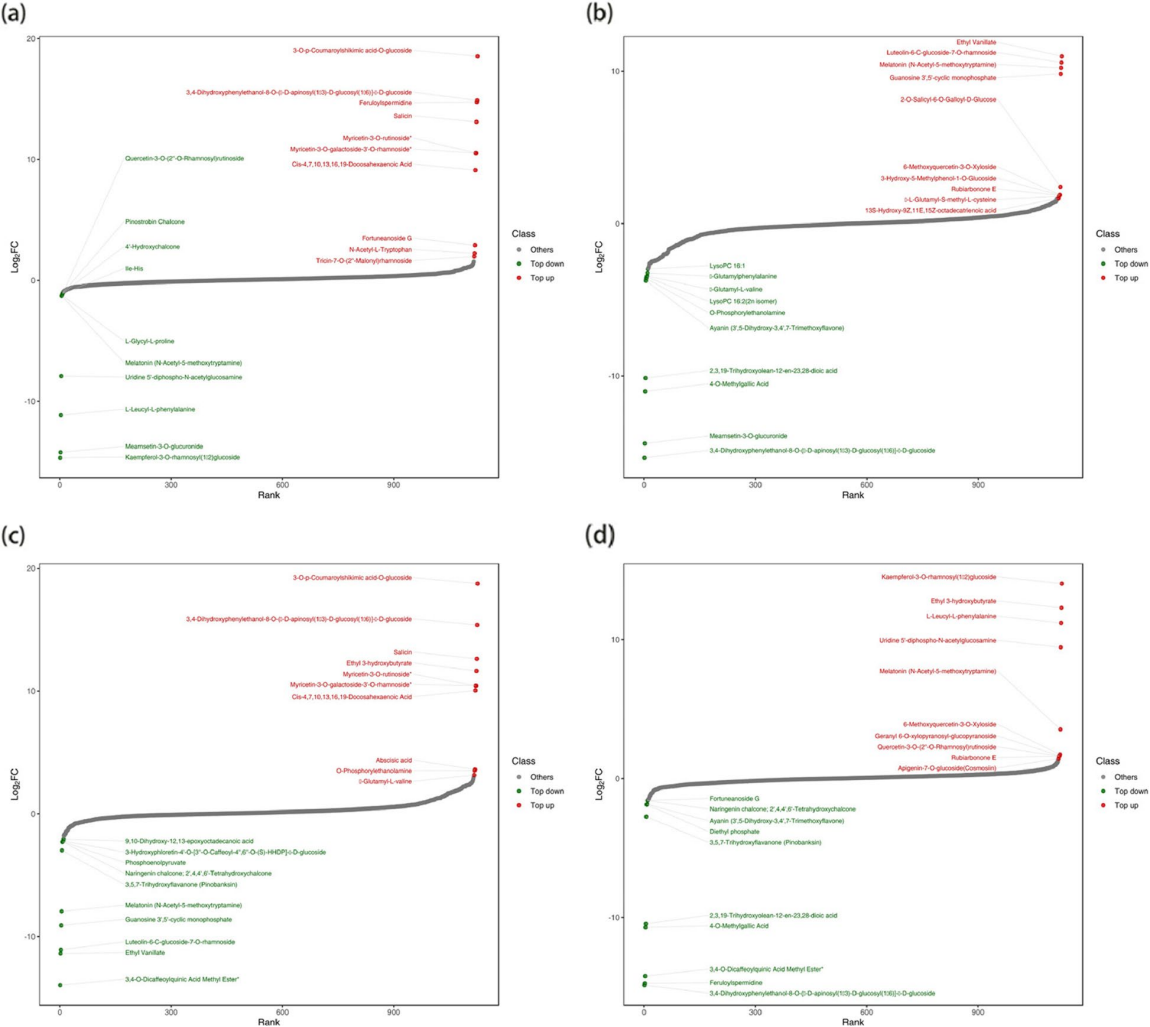
Difference multiple of metabolites among groups: **a** Top 20 metabolites with large differences in OE-CK_vs_OE-D group, **b** Top 20 metabolites with large differences in WT-CK_vs_OE-CK group, **c** Top 20 metabolites with large differences in WT-D_vs_OE-D group. **d** Top 20 metabolites with large differences in WT-CK_vs_WT-D group

Figure 9 shows the magnitude of metabolite differences between different combinations of groups, and the top 20 metabolites with substantial differences between groups are listed (a total of 50 species). A total of 13 metabolites were annotated to Metabolic pathways, which were uridine diphosphate acetylglucosamine, melatonin (N-acetyl-5-methoxytryptamine), apigenin-7-O-glucoside, naringenin chalcone, ayanin (3’, 5-dihydroxy-3,4’,7-trimethoxyflavone), cis-4,7,10,13,16,19-docosahexaenoic acid, salicylin, abscisic acid, phosphoethanolamine, phosphoenolpyruvate, 3,5,7-trihydroxyflavanone (pinostrobin), guanosine 3’, 5’ -cyclic monophosphate, 13S-hydroxy-9Z, 11E, 15Z-octadecadienoic acid, and 2’ -deoxyinosine-5’-monophosphate.

A total of 155 DAMs were enriched in pathways in non-transgenic plants in the normal-watered and drought treatment groups, accounting for 13.8% of the DAMs (1,127), which were distributed in 76 metabolic pathways. A total of 42 DAMs were enriched in Metabolic pathways (ko01100), 29 differentially accumulated metabolites were enriched in Biosynthesis of secondary metabolites (ko01110), and 13 DAMs were enriched in Biosynthesis of amino acids (ko01230). There were 10 DAMs involved in Aminoacyl-tRNA biosynthesis (ko00970) and 10 DAMs involved in Biosynthesis of cofactors (ko01240) (Supplementary Table S9-S10).

**Fig. 10:**
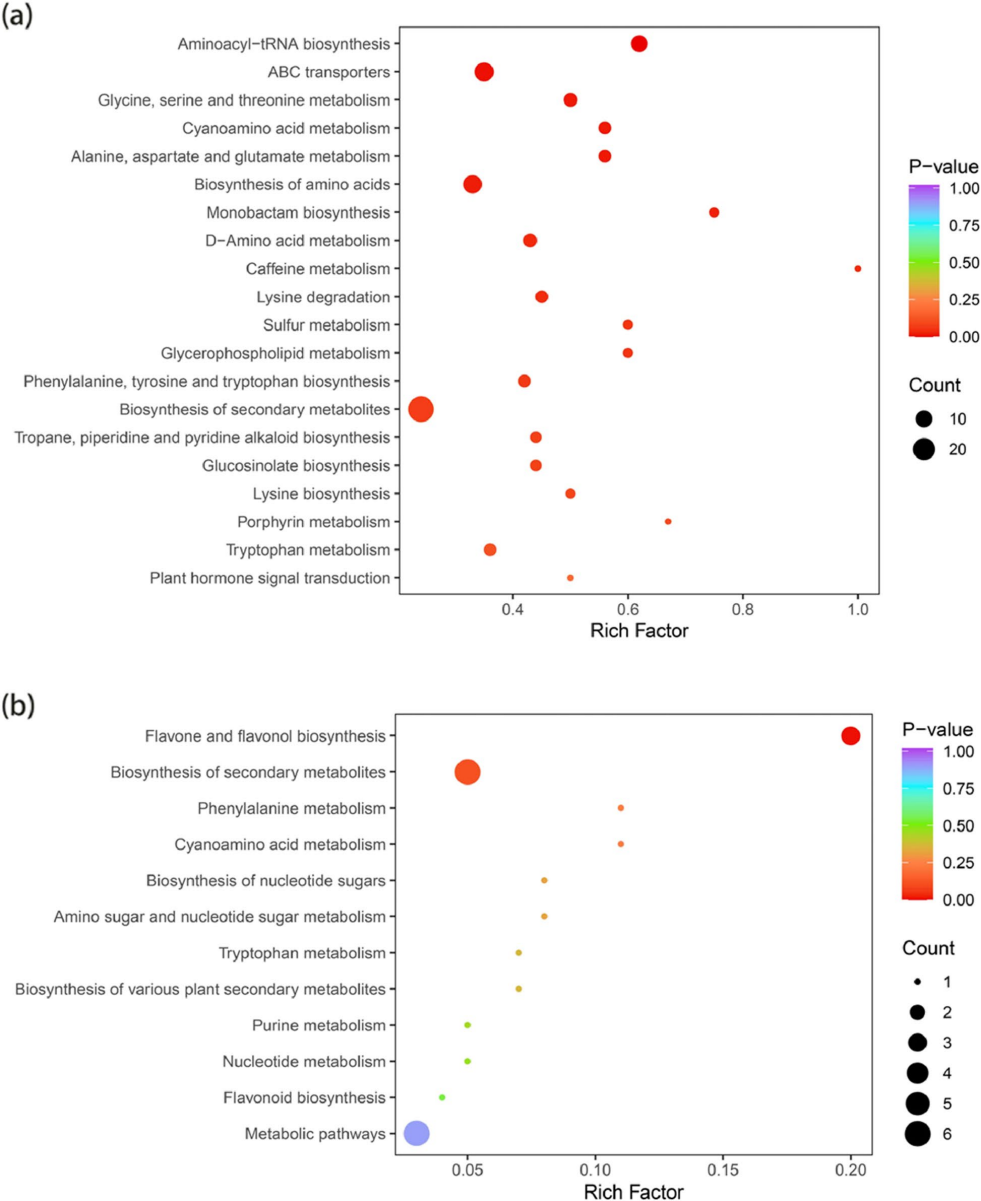
Map of pathways with DAMs and KEGG enrichment map: **a** KEGG enrichment map of non-transgenic plants in normal-watered and drought-treated groups, **b** KEGG enrichment map of transgenic plants in normal-watered and drought-treated groups

Thirty DAMs, representing 2.7% of all DAMs (1,127), were enriched in 12 metabolic pathways in transgenic plants in the normal-watered and drought-treated groups. Six DAMs involved in Metabolic pathways (ko01100), 6 DAMs involved in Biosynthesis of secondary metabolites (ko01110), and 3 DAMs involved in Flavone and flavonol biosynthesis (ko00944) were identified (Fig. 10).

A total of 44 metabolites were significantly up-regulated in Metabolic pathways in non-transgenic plants in the normal-watered and drought-treated groups, 16 Amino acids and derivatives, L-Glutamine, L-Lysine, L-Aspartic acid, N6-Acetyl-L-lysine, L-Tryptophan, S-Methyl-L-cysteine, L-Valine, L-Proline, L-Threonine, L-Homoserine, L-Phenylalanine, L-Tyrosine, Oxiglutatione, O-Acetyl serine, Trimethyllysine, and L-Asparagine; 2 Phenolic acids (Salicin and Trans-5-O-(p-Coumaroyl)shikimate); 6 Nucleotides and derivatives (Flavoside, Uridine 5’-diphospho-N-acetylglucosamine, β-Nicotinamide mononucleotide, Xanthine, Uridine, and Cytidine); 2 Flavonoids, (Apigenin-7-O-glucoside (Cosmosiin); Ayahuasca); 2 others (Raffinose and Pyridoxine); 7 Alkaloids (O-Phosphorylethanolamine Methoxyindoleacetic acid, Indole, 3-Indoleacetonitrile, Piperidine, 5-Aminopentanoic acid, and Choline); 6 Organic acids (ABA, Succinic acid, 6-Aminohexanoic acid, Methylmalonic acid, Gamma-aminobutyric acid, and L-Piperidinic acid); and 3 Lipids (Cis-4,7,10,13,16,19-Docosahexaenoic acid, Glyphosphorylcholine GPC, and 9-Hydroxy-12-oxo-10(E),15(Z)-octadecadienoic acid (Supplementary Table S9). The response of non-transgenic plants to drought stress is associated with the accumulation of these metabolites.

A total of four metabolites were significantly up-regulated in the metabolic pathways of transgenic plants in the normal-watered and drought-treated groups, including 2 Alkaloids (Amygdalin and Melatonin); 1 Flavonoid (Apigenin-7-O-glucoside (Cosmosiin)); and 1 Nucleotide and derivative (Uridine 5’-diphospho-N-acetylglucosamine) (Supplementary Table S10). The response of transgenic plants to drought stress is associated with the accumulation of Alkaloids, Nucleotides and derivatives, Flavonoids, and other substances. Amygdalin was only up-regulated in transgenic plants, Melatonin was up-regulated in transgenic plants and down-regulated in non-transgenic plants, and Apigenin-7-O-glucoside and Uridine 5’-diphospho-N-acetylglucosamine were more significantly up-regulated in transgenic plants.

### KEGG co-enrichment analysis of DEGs and DAMs

**Fig. 11:**
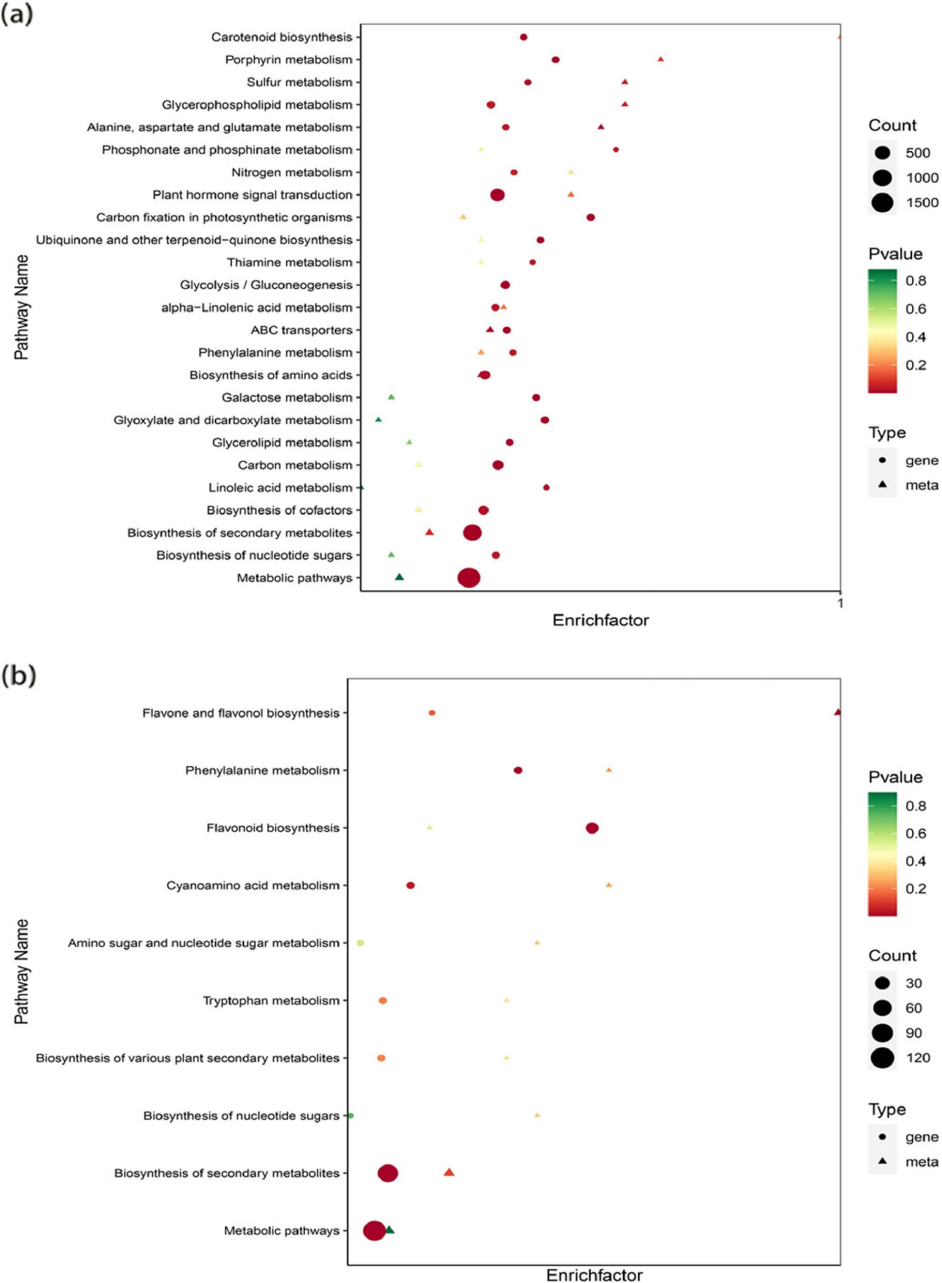
Co-enrichment of DAMs (differentially accumulated metabolites) and DEGs (differentially expressed genes): **a** DAM and DEG co-enrichment betweeen drought-treated and normal-watered treatment groups in non-transgenic plants, **b** Patterns of DAM and DEG co-enrichment between the drought-treated and normal-watered treatments groups in transgenic plants

Pathway co-enrichment analysis of DEGs and DAMs in non-transgenic plants in the drought treatment and normal-watered treatment groups was conducted. The results revealed 50 co-enriched metabolic pathways and six DAMs (*P*-value < 0.05) involved in the metabolism of ABC transporters; glycine, serine and threonine metabolism; metabolism of alanine, aspartate and glutamate; biosynthesis of amino acids; biosynthesis of monolactam; and lysine degradation. There were 20 DEGs (*P*-value < 0.05), that were involved in the metabolism of galactose; metabolic pathways; biosynthesis of secondary metabolites; glycolysis/gluconeogenesis; biosynthesis of ubiquinone and other terpenoids-quinones; biosynthesis of carotenoids; ABC transporters; glycerolipid metabolism; biosynthesis of amino acids; sulfur metabolism; linoleic acid metabolism; biosynthesis of cofactors; α-linolenic acid metabolism; alanine, aspartate and glutamate metabolism; thiamin metabolism; nucleotide glycoside biosynthesis; phosphate and phosphate metabolism; phenylalanine metabolism; glycerophospholipid metabolism; and nitrogen metabolism (Fig. 11a).

Pathway co-enrichment analysis of DEGs and DAMs in transgenic plants in the drought treatment vs. Normal-watered treatment groups was conducted. Ten co-enriched metabolic pathways were identified; one DAM (*P*-value < 0.05) involved in the biosynthesis of flavonoids and flavonols was identified. There were five DEGs (*P*-value < 0.05) involved in metabolite pathways, secondary metabolite biosynthesis pathway, phenylalanine metabolism, cyanogenic amino acid metabolism, and flavonoid biosynthesis. Flavonoid biosynthesis, cyanogenic amino acid metabolism, and metabolic pathways were only enriched in transgenes (Fig. 11b).

### Differences in DEG and DAM expression in the flavonoid biosynthesis pathway between transgenic and non-transgenic plants

**Fig. 12:**
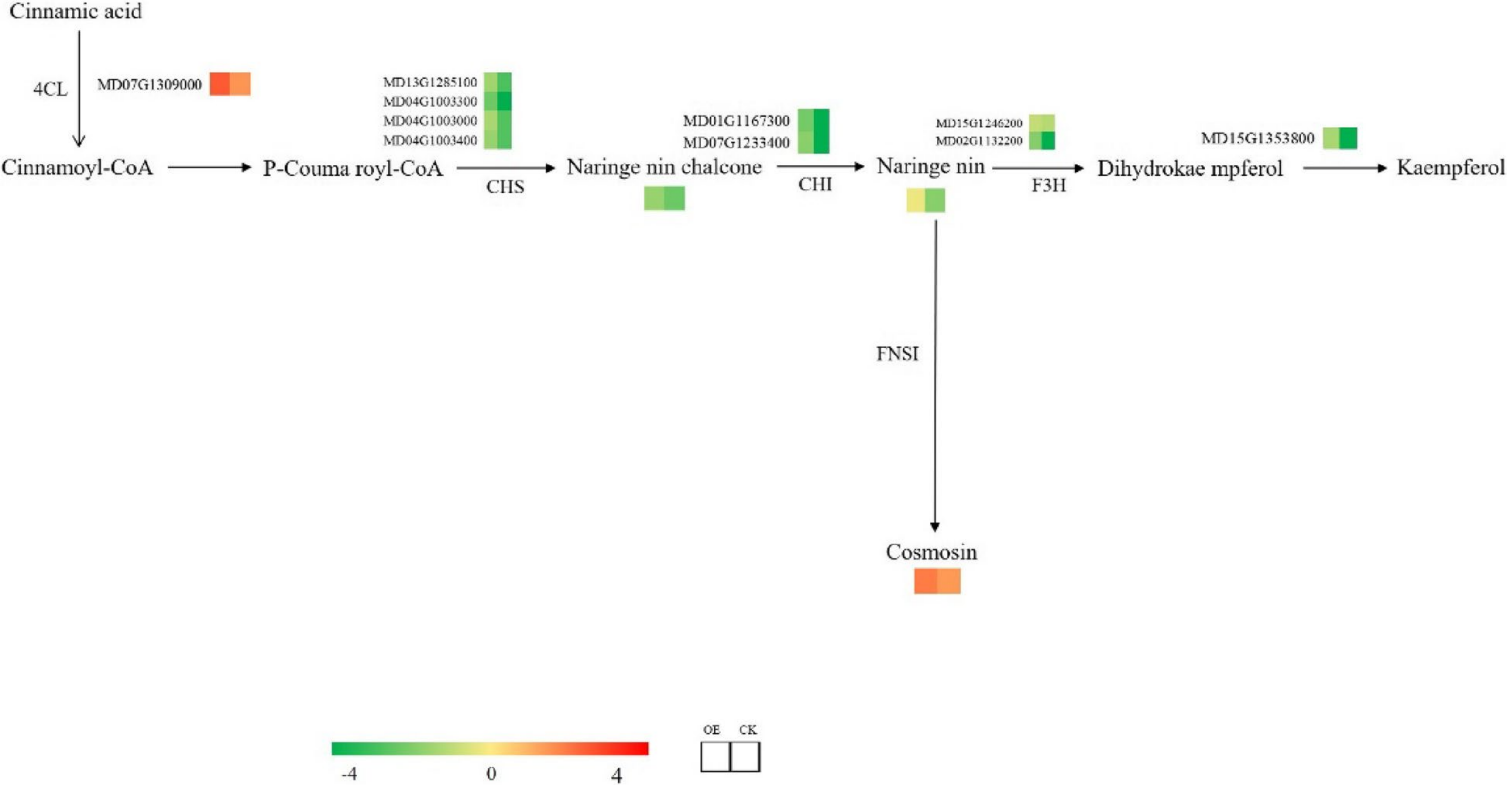
Heat map of flavonoid metabolic pathways under drought stress in transgenic and non-transgenic plants

 Under drought stress, naringenin chalcone was down-regulated in both transgenic and non-transgenic plants, but it was less down-regulated in transgenic plants (Fig. 12). The naringenin content was not significantly altered in transgenic plants and down-regulated in non-transgenic plants; the apigenin-7-O-glucoside content was up-regulated in both transgenic and non-transgenic plants but was more significantly up-regulated in transgenic plants. The expression of the genes encoding CHS and CHI was down-regulated in both transgenic and non-transgenic plants; the down-regulation of these genes was more significant in non-transgenic plants, and the expression of *4CL* genes was up-regulated more significantly in transgenic plants.

## Discussion

### Effect of drought stress on physiological and biochemical indicators of transgenic and non-transgenic apple plants

Drought stress induces damage to the chloroplast membrane, which destroys the structure of the chloroplast and reduces the activity of photosystem II (PSII); this results in the production of a large amount of ROS and destabilizes the cell membrane, which inhibits photosynthesis in plants. Chlorophyll is an important photosynthetic pigment that plays an important role in the process of light energy absorption, transmission, and conversion. Studies have shown that chlorophyll is damaged during drought stress, which reduces the chlorophyll content in cells [33]. Drought stress limits plant growth by reducing the Pn [34]. The Pn of tomato decreases as the degree of drought stress increases [35]. In our experiment, *MdPYL9*-overexpressing plants and non-transgenic plants were subjected to short-term natural drought treatment. The Pn, Gs, Tr, Ci, and chlorophyll content of transgenic plants did not significantly differ with non-transgenic plants under normal-watered conditions. Under drought treatment, the Pn, Gs, and Tr of all plants decreased, and the Ci concentration of all plants increased. The Pn, Gs, and Tr of transgenic apple plants under drought stress treatments decreased to a lesser extent, the Ci concentration increased to a lesser extent compared with the non-transgenic plants. The chlorophyll contents of both non-transgenic and transgenic apple plants were significantly reduced, but the decrease of transgenic plants was significantly less than that of non-transgenic ones.This might stem from the fact that transgenic plants were less damaged by drought stress and had higher PSII activity compared with non-transgenic plants.

Drought stress inhibits plant growth and development; alters plant morphology; affects physiological, biochemical, and metabolic characteristics; and is detrimental to plant growth [36]. This causes the excessive accumulation of ROS in plants, which leads to cell membrane peroxidation, MDA production, changes in membrane permeability, electrolyte leakage, and increased ion permeability; however, the antioxidant system can scavenge ROS [37, 38]. After 6 d of drought stress, the wilting was severe in non-transgenic plants and weak in transgenic plants; after 7 d of rehydration, non-transgenic apple plants dried and died, and transgenic apple plants recovered. The REL and content of MDA, O_2_^−^, and H_2_O_2_ in transgenic and non-transgenic apple plants significantly increased after drought stress, but the transgenic lines accumulated significantly less contents of MDA, O_2_^−^, and H_2_O_2_ and less REL than non-transgenic plants. After 6 d of drought stress, the SOD, POD and CAT activities of both non-transgenic and transgenic plants decreased significantly, but the SOD and CAT activities of transgenic plants were significantly higher than those of non-transgenic plants. The overexpression of *MdPYL9* can help maintain cellular homeostasis and alleviate the extent of cellular damage under stress. These physiological indexes were consistent with the observed phenotype under drought stress, indicating that overexpression of *MdPYL9* enhanced the resistance of apple plants to drought stress.

### Transcriptomic analysis of drought-stressed transgenic and non-transgenic apple plants

We concluded that overexpression of *MdPYL9* gene in apple enhanced the drought tolerance of apple plants. The molecular mechanisms underlying the plant drought response have been revealed using molecular biotechnology [39]. External drought stimuli are sensed by unknown sensors on the membrane, and the signals are then transmitted through multiple signaling pathways, leading to the expression of drought-responsive genes that confer drought tolerance in plants [40, 41]. RNA-seq has been widely used to unravel the molecular basis of the drought response in many plant species [42–47]. We performed transcriptome sequencing to further clarify the role that *MdPYL9* plays in the drought stress response. The results showed that transgenic plants had 334 up-regulated genes and 174 down-regulated genes under normal-watered and drought conditions. A total of 7,153 genes were up-regulated and 6,400 genes were down-regulated in control plants under normal and drought conditions. Fewer DEGs responded to drought stress in transgenic plants than in non-transgenic plants. This indicates that *MdPYL9*-overexpressing plants were less sensitive to drought than non-transgenic plants, and reductions in the number of DEGs have been reported in other drought-tolerant plant species [44, 48, 49]. This might stem from the lack of homeostatic mechanisms in susceptible genotypes to mitigate the effects of drought. Based on changes in the number of DEGs in the transcriptome, *MdPYL9* plays a role in regulating the response to drought stress.

In the GO functional classification, the GO entries enriched under drought conditions were the same as those under normal treatment conditions, but 7 biological process entries and 7 molecular function entries were enriched under drought, and these GO entries were enriched under drought conditions. More differentially expressed genes under drought, indicating that drought stress leads to a series of stimulus responses in apple seedlings. The GO entries of differential gene annotation in transgenic lines can be found in the GO entries of differential gene annotation in non-transgenic lines, and there are 6 more biological process entries, 6 more molecular function entries, and 6 more than the GO entries of transgenic lines. The cell component entries of differential gene annotation are the same, but after drought stress, the number of genes whose expression changes under the two entries of the non-transgenic is more. It may be due to under drought stress, the non-transgenic plants showed drought resistance, wilting degree is more serious, causing changes in other physiological and biochemical processes, as well as changes in the activity of small molecules such as enzymes, resulting in the increase of differential genes, resulting in the increase of GO functional items annotated by differential genes. By observing the significance of KEGG metabolic pathway enriched by differential genes, the tolerance of apple to drought stress can be judged. In this experiment, compared with normal water supply, under drought stress, in the KEGG pathway enriched by differential genes in transgenic and non-transgenic lines, the differentially expressed genes in non-transgenic plants were mainly enriched in the plant hormone signal transduction pathway, and the differentially expressed genes in transgenic plants were mainly enriched. The pathway is the flavonoid biosynthesis pathway. Plant hormone signaling pathways are involved in abiotic stress adaptation through ubiquitin-mediated protein hydrolysis or ABA-mediated responses [50, 51]. Flavonoids are non-enzymatic protective substances that can effectively scavenge ROS and improve the resistance of plants to drought stress. Many studies have shown that the accumulation of flavonoids and the expression of flavonoid biosynthesis genes increase under drought stress [52–54]. It indicated that in this experiment, the flavonoids related to drought stress in transgenic plants began to be processed and synthesized, thereby enhancing the ability of transgenic plants to scavenge ROS, while the non-transgenic plants remained in the signal transduction response to drought stress stage, indicating that the non-transgenic plants responded slowly to drought. In summary, the *MdPYL9* gene may promote the synthesis of a certain flavonoid, which in turn improves the drought tolerance of transgenic plants.

### Metabolomic analysis of transgenic and non-transgenic apple plants under drought stress

The metabolome of higher plants consists of thousands of primary and secondary metabolites, and approximately 10% of these metabolites have been identified to date [55]. Metabolites mediate responses to environmental changes [56] (Lu et al. 2013). Metabolomics can be used to quantitatively and qualitatively characterize changes in metabolites in plants under stress, and this information can be used to link genotype and phenotype [57, 58]. Many metabolites are altered under drought stress, such as soluble sugars, organic acids, phenols, amino acids, fatty acids, nucleotides, and secondary metabolites, and these are important components of the plant defense system [44, 59–61]. In Arabidopsis, the levels of most amino acids, TCA cycle intermediates, flavonoids, and lipids were elevated under drought stress [62–64]. Similar changes were observed in rice and maize [44, 65, 66].

In this experiment, 155 metabolites were detected in non-transgenic plants and in transgenic plants; 30 metabolites were detected after drought stress, indicating that *MdPYL9*-overexpressing plants were less sensitive to drought than non-transgenic plants. A lesser increase in differential metabolites in drought-tolerant varieties has also been reported in studies of wheat [67]. A total of 4 metabolites, were significantly up-regulated in the metabolic pathways of transgenic plants. A total of 44 metabolites were significantly up-regulated in the metabolic pathways of non-transgenic plants.Bitter amygdalin and melatonin were only up-regulated in transgenic plants. Apigenin-7-O-glucoside and Uridine 5’-diphospho-N-acetylglucosamine were more significantly up-regulated in transgenic plants, which might reflect differences in the response mechanism to drought stress between them, with apigenin-7-O-glucoside being the most correlated with the drought response in transgenic plants and ABA being the most correlated with the drought response in non-transgenic plants.

### Joint transcriptomic analysis of the metabolome of drought-stressed transgenic and non-transgenic apple plants

Flavonoids are important secondary metabolites in plants; they are widely stored in plants and play a key role in various biochemical and physiological processes of plants [68–70]. Several studies have shown that flavonoids are ROS scavengers [71–73]. They protect plants from various abiotic stresses and act as a unique UV filter, antioxidant, and even as signaling molecule [74]. In soybean, flavonoids accumulate and attenuate the effects of UV and drought stress [75]. In Arabidopsis, the accumulation of flavonoids determines freezing tolerance [76]. In rice, overexpression of the *SQD2.1* gene enhances salt and drought resistance in rice, and the flavonoid content increases in *SQD2.1*-overexpressing rice plants and decreases in *SQD2.1* rice mutants, especially apigenin-7-O-glucoside [77]. The flavonoid apigenin enhances tolerance to salt stress in rice by increasing the chlorophyll content and total flavonoid levels while reducing Na + accumulation in the root system [78]. Apigenin-7-O-glucoside is a derivative of apigenin, and the content of apigenin-7-O-glucoside in rice was elevated by drought stress [79]. *CHS* encodes the first enzyme in the flavonoid pathway and CHI is the key enzyme in the flavonoid biosynthesis pathway; CHI converts chalcone to naringin [80]. The *4CL* gene plays an important role in a key bifurcation point in the biosynthesis of compounds such as flavonoids. 4CL, CHI, and CHS are key enzymes in the apigenin synthesis pathway [81]. In our study, the expression of *CHS* and *CHI* was down-regulated in both transgenic and non-transgenic plants, but *CHS* and *CHI* were more significantly down-regulated in non-transgenic plants; the expression of *4CL* was up-regulated in both transgenic and non-transgenic plants, but it was more significantly up-regulated in transgenic plants, which in turn led to a decrease in the naringin chalcone content in both transgenic and non-transgenic plants. Changes in the naringin content were not significant.

## Conclusion

It indicated that the overexpression of *MdPYL9* increased the drought resistance of plants by up-regulating the expression of *4CL* gene, down-regulating the expression of *CHS* and *CHI* genes, and then increasing the content of apigenin-7-O-glucoside under drought stress. Thus, drought resistance of apple plants could be improved.

### Supplementary Information

Supplementary Material 1: Supplementary Table S1. qRT-PCR primers in this study.

Supplementary Material 2: Supplementary Table S2. Transcriptome sequencing data and quality assessment.

Supplementary Material 3: Supplementary Table S3. Differentially expressed genes in the OE-CK_vs_OE-D comparison.

Supplementary Material 4: Supplementary Table S4. Differentially expressed genes in the WT-CK_vs_WT-D comparison.

Supplementary Material 5: Supplementary Table S5. Differentially expressed genes in the WT-CK_vs_OE-CK comparison.

Supplementary Material 6: Supplementary Table S6. Differentially expressed genes in the WT-D_vs_OE-D comparison.

Supplementary Material 7: Supplementary Table S7. Number of DEGs annotated to enriched KEGG pathways for control plants.

Supplementary Material 8: Supplementary Table S8. Number of DEGs annotated to enriched KEGG pathways for transgenic plants.

Supplementary Material 9: Supplementary Table S9. Up-regulated metabolites involved in the metabolic pathways of control plants in the normal and drought-treated groups in response to drought stress.

Supplementary Material 10: Supplementary Table S10. Up-regulated metabolites in the metabolic pathways of normal and drought-treated groups of transgenic plants in response to drought stress.

Supplementary Material 11: Supplementary Table S11-S13. Physiological, Transcriptomic and Metabolome raw data.

## Data Availability

Data supporting this study are included within the article and/or supporting materials. The datasets generated and analysed during the current study are available in the Sequence Read Archive (SRA) repository at the National Center for Biotechnology Information (NCBI) under the accession number PRJNA1066410.
